# Challenges in reducing the 10-item CARE Measure to a two-item version: comparison of patients’ preferences with psychometric evaluation in a cross-sectional survey in Scotland

**DOI:** 10.3399/BJGPO.2025.0085

**Published:** 2026-02-11

**Authors:** Lauren Ng, Kieran D Sweeney, Stewart W Mercer

**Affiliations:** 1 Usher Institute, University of Edinburgh, Edinburgh, UK

**Keywords:** empathy, psychometrics, survey, patient preference, general practice

## Abstract

**Background:**

The Consultation and Relational Empathy (CARE) Measure is a widely used 10-item measure to assess patients’ perceptions of physician empathy. Takahashi *et al*’s (2022) recent study proposed a two-item version based on psychometric evaluation of survey responses, without considering patient preferences.

**Aim:**

To apply Takahashi *et al*’s psychometric method to UK data, and compare findings with patients’ preferences on the two most important items.

**Design & setting:**

In 2022, a cross-sectional postal survey of 6291 Scottish adults was conducted.

**Method:**

Using Takahashi *et al*’s method, psychometric evaluation compared correlations between all possible two-item combinations with the original 10-item CARE Measure to identify the optimal two-item combination. Patients were also asked to select the two items they considered most important. Descriptive analysis examined the proportion of patients selecting each item, and level of agreement on the most popular two-item combination.

**Results:**

In total, 1053 (17%) of 6291 patients responded. Psychometric evaluation identified items 6 (‘Showing care and compassion’) and 8 (‘Explaining things clearly’) as the optimal two-item combination (Cronbach’s alpha = 0.916, correlation = 0.953). This differed from patient preferences, with items 3 (‘Really listening’) and 8 receiving the highest proportion of votes (19% and 17%, respectively). Preferences also varied by age, deprivation level, and consultation complexity. The most popular two-item combination (items 3 and 8) was selected by 10% of responders, with 90% selecting other combinations.

**Conclusion:**

The psychometrically optimal two-item combination did not align with patient preferences. Given variation in patient preferences and low agreement, reducing the CARE Measure to two-items may be inadvisable.

## How this fits in

The CARE Measure is a widely used tool for assessing patients’ perceptions of physician empathy. Takahashi *et al* (2022) proposed a two-item version based on psychometric analysis, but this approach did not account for patient preferences. Applying this psychometric method to UK data, our study found that the psychometrically optimal two items differed from Takahashi *et al*’s (2022) two-item selection and those most valued by patients, whose preferences also varied by age, deprivation, and consultation complexity. These findings suggest caution in reducing the CARE Measure and highlight the importance of incorporating patient views in measure design.

## Introduction

Physician empathy is widely regarded as a core aspect of high-quality interpersonal care and is known to improve a range of patient outcomes.^1,2^ It is a complex, multidimensional construct that integrates cognitive, emotional, moral, and behavioural elements, and involves the capacity to understand a patient’s perspective, communicate that understanding effectively, and respond in a therapeutically meaningful way.^1^ The Consultation and Relational Empathy (CARE) Measure is a widely used patient-rated experience measure designed to evaluate patients’ perceptions of physician empathy.^3^ It comprises 10 items that have been extensively validated across numerous countries and healthcare settings.^4–6^

Recently, a two-item version of the CARE Measure was developed in Japan by Takahashi *et al* ^7^ consisting of item 6 (‘Showing care and compassion’) and item 9 (‘Helping you to take control’) from the original 10-item measure. This abbreviated version was designed to reduce completion time and responder burden in time-pressured clinical settings.^7^ The selection was based on the psychometric evaluation of responses to the CARE Measure in a survey of 252 patients in Japan. This involved identifying the shortest combination of items with a Cronbach alpha of >0.90, which had the highest correlation with the original 10-item version. The combination of items 6 and 9 was identified as the optimal abbreviation, and was then approved by a team of physicians and researchers.^7^ Although the abbreviated tool may offer some practical benefits, the absence of patient involvement in its development raises concerns as to whether it adequately reflects the multidimensional nature of physician empathy.

There has been increasing recognition of the importance of incorporating patient views in health research, including in survey development.^8^ This ensures that patients’ expectations, needs, and preferences are addressed; thus, leading to more relevant and effective tools.^9^ The necessity of patient views is further reflected in both the COSMIN guidelines (COnsensus-based Standards for the selection of health Measurement INstruments)^10^ and the minimum standards set out by the International Society for Quality of Life Research (ISOQOL).^11^

Therefore, this study aims to:

1. identify which two-item combination would be selected in the UK, using the same psychometric criteria as Takahashi *et al*;^7^
2. determine which two items patients consider most important, and whether this varies according to individual and consultation characteristics; and
3. assess the level of agreement among patients regarding the most popular two-item combination.

## Method

### Study design

This cross-sectional study comprised a postal survey of 6291 adult patients, conducted as part of a wider evaluation of the impact of the 2018 GP contract on health inequalities in Scotland.^12^

### Sampling, recruitment, and data collection

A detailed description on sampling, recruitment, and data collection methods are provided in full elsewhere.^12^ In brief, a purposive sample of 12 GP practices was recruited from three regional health boards in Scotland (four in each region) to reflect a range of geographic and socioeconomic characteristics, including health boards with the highest and lowest levels of deprivation. The study design was not intended to be representative of the general population, but rather to give a range of patient characteristics for those who had recently consulted a GP. Differences between responders and non-responders have also been reported previously. Overall, responders tended to be older than non-responders, with responders in the high-deprivation urban (HDU) group being significantly less deprived.^12^

Within each practice, a random sample of 6291 adults (aged≥18 years) who had consulted a GP within the past 30 days was identified from practice records. The sample size was based on the authors’ prior research, which showed statistically significant differences between affluent and deprived practices,^13,14^ with oversampling in more deprived areas where lower response rates were anticipated.^12^ Recruitment targets were also limited by the study’s time and resource constraints.

Questionnaires were distributed with a cover letter and participant information sheet, and returned through pre-paid envelopes. Owing to time and funding limitations, no reminders were sent. Data collection took place from 31 August to 30 November 2022.

### Consent and patient and public involvement

All participants received a study information sheet, and return of a completed questionnaire was taken as implied consent. Patient and public involvement informed all aspects of the original survey design,^12^ but were not involved in the preparation of this current manuscript, as the study funding had concluded before its development.

### Instruments used

The survey collected data on patient and consultation characteristics, as previously described in full.^12^ Demographic variables included age, sex, deprivation status, and rurality.^12,15^ A blank copy of the full survey questionnaire is provided in Supplementary Box S1.

GP practices were grouped by geographic area, based on whether they served mainly remote and rural (RR), HDU, or low-deprivation urban (LDU) areas.

Consultation characteristics included the number of problems and types of problems discussed (physical, psychological, social, administrative, or other). Consultations were defined as complex if they involved a physical problem plus a psychological and/or social problem.^16^

**Figure 1.**
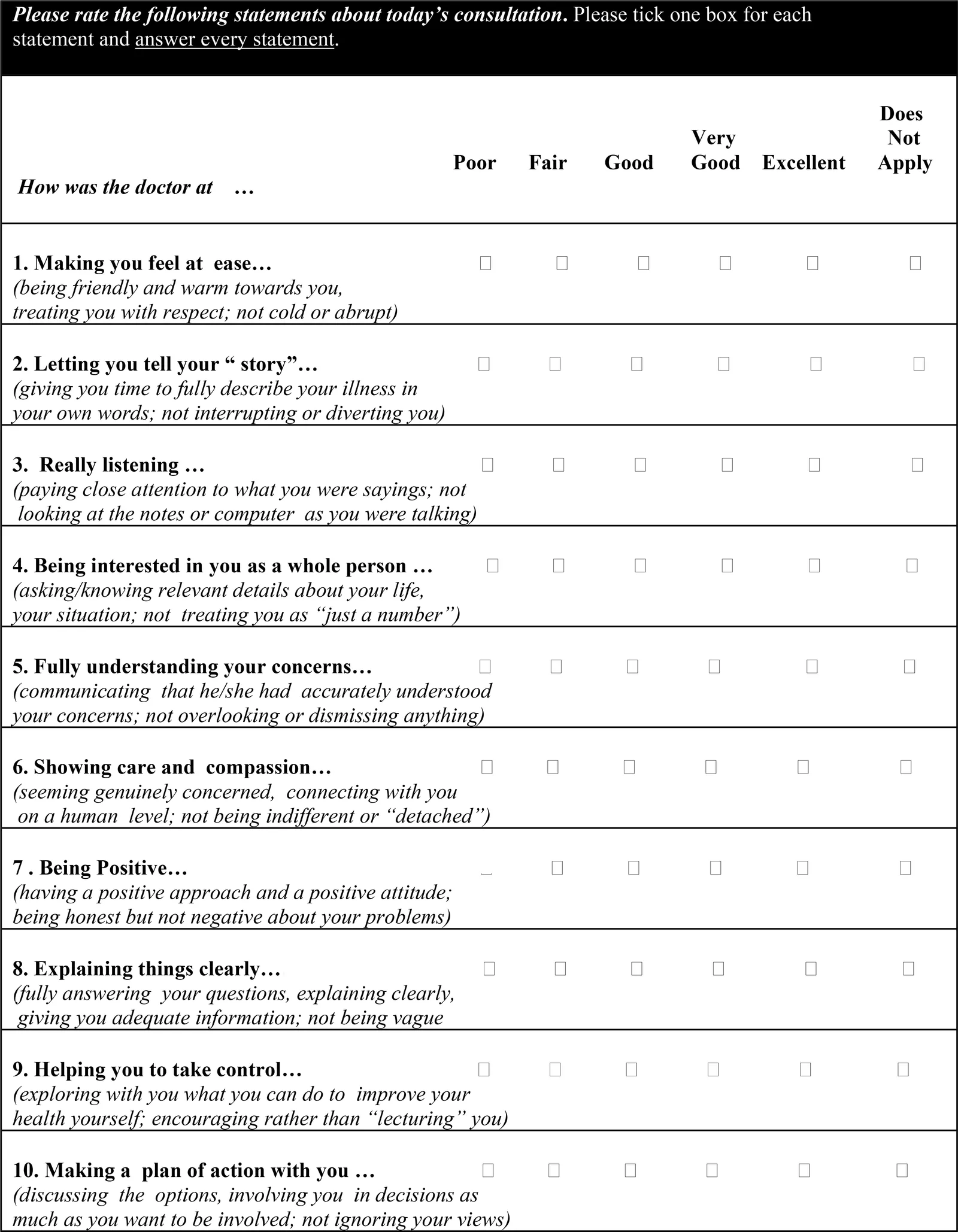
The 10 items in the Consultation and Relational Empathy (CARE) Measure

Perceived GP empathy was evaluated using the 10-item CARE Measure (Figure 1).^3^ Additionally, patients were asked to select the two items from the CARE Measure they considered ‘most important to them’ when seeing their GP.^12^

### Data analysis

First, psychometric evaluation of CARE Measure responses was performed using the *psych* package in R, following the methodology of Takahashi *et al.* ^7^ This involved evaluating the internal reliability consistency (Cronbach alpha) for all 1053 possible abbreviated item combinations (from two-item versions up to nine-item versions) and assessing the correlation between these abbreviated versions and the original 10-item measure. A selection was then made based on the following criteria:

1. the shortest combination with a Cronbach alpha of >0.90; and
2. the combination with the highest correlation with the 10-item version.

Second, patient responses regarding the two most important items were analysed descriptively on SPSS (version 27). The proportion of all votes for each individual item was assessed, enabling all 10 items to be ranked in order of patient preference. Variations across individual and consultation characteristics were examined. Following this, the level of agreement regarding the most popular two-item combination was assessed by calculating the proportion of responders selecting each of the 45 possible two-item combinations.

No imputation was performed for missing data. Responses with no votes were excluded from these descriptive analyses. Responses with only one vote (where responders only selected one item in response to the question ‘Which two items are most important to you?’) were still included in the ranking of individual items; whereas responses with two votes were included in the analysis of agreement regarding two-item combinations.

## Results

In total, 1053 out of 6291 patients responded to the survey (17% response rate). The consultation and patient characteristics are shown in Supplementary Table S1. The mean age was 62.8 years, with 60% female responders, and 43% from HDU areas. Most consultations were face to face (63%) or via telephone (36%).

The original 10-item CARE Measure is shown in Figure 1. Each responder (*n* = 1053) was asked to select the two CARE Measure items they considered most important. Of the 2106 expected item selections, 143 selections (7%) were missing, as some responders selected only one item or none.

### Psychometric analysis

Psychometric analysis found that the majority of two-item combinations (29 out of 45) had a Cronbach’s alpha >0.90. Therefore, in line with Takahashi *et al*’s study,^7^ a two-item version was preferred to a longer abbreviation (those containing 3–9 items). The combination of items 6 and 8 demonstrated the highest correlation with the original 10-item version (Cronbach’s alpha = 0.916, correlation of 0.953). By contrast, the two-item combination selected by Takahashi *et al* (items 6 and 9) had a correlation of 0.809 with the original version, making it the 24^th^ ranked two-item combination based on this criteria.

### Individual item ranking

**Figure 2.**
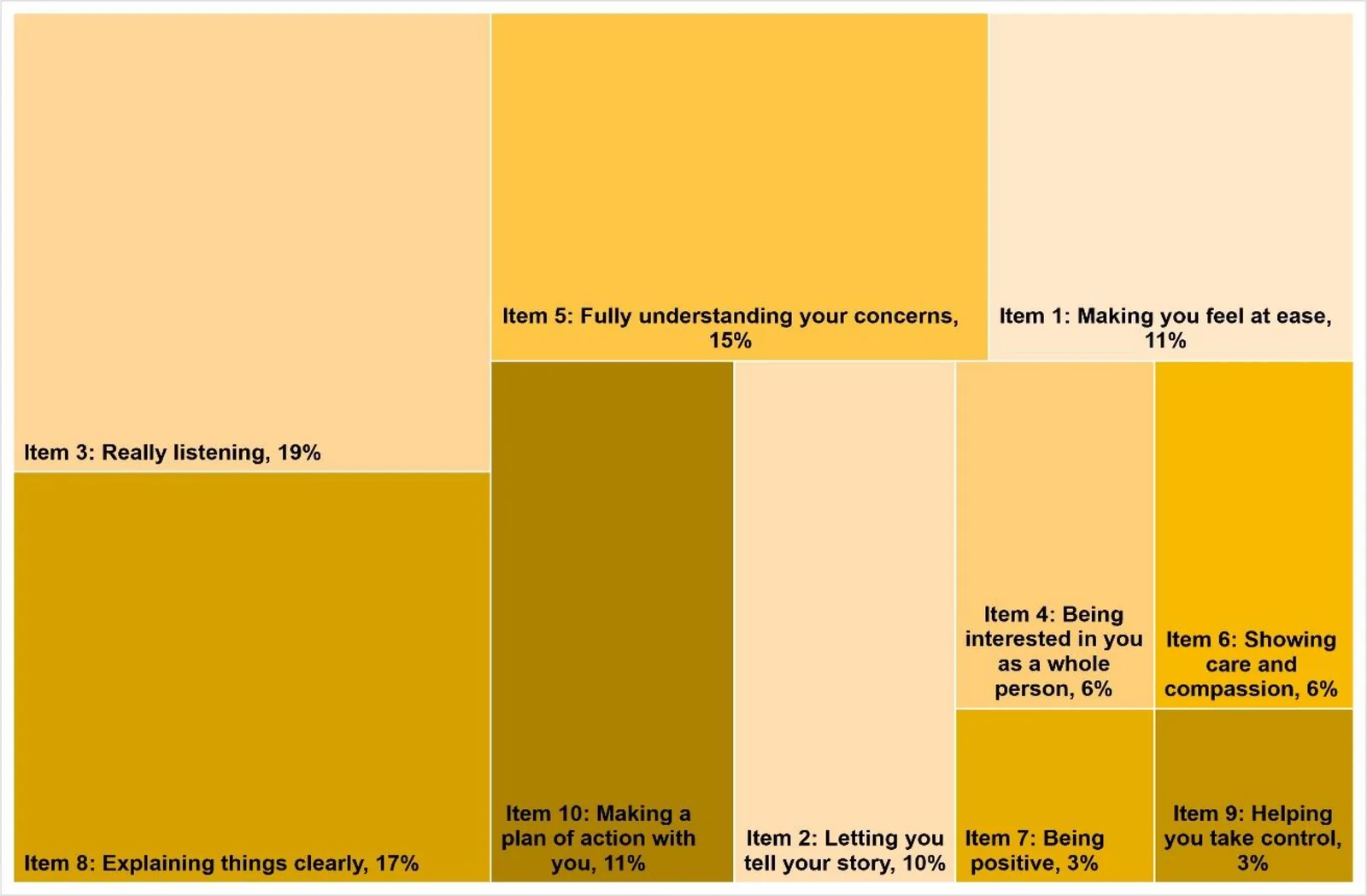
Tree diagram illustrating the proportion of votes for each of the 10 CARE Measure items overall

When aggregating all votes (two per patient) for the most important items, item 3 (‘Really listening’) was ranked highest with 19% of votes, followed by item 8 (‘Explaining things clearly’) with 17% of votes. The proportion of votes for all 10 items is shown in Figure 2.

**Figure 3.**
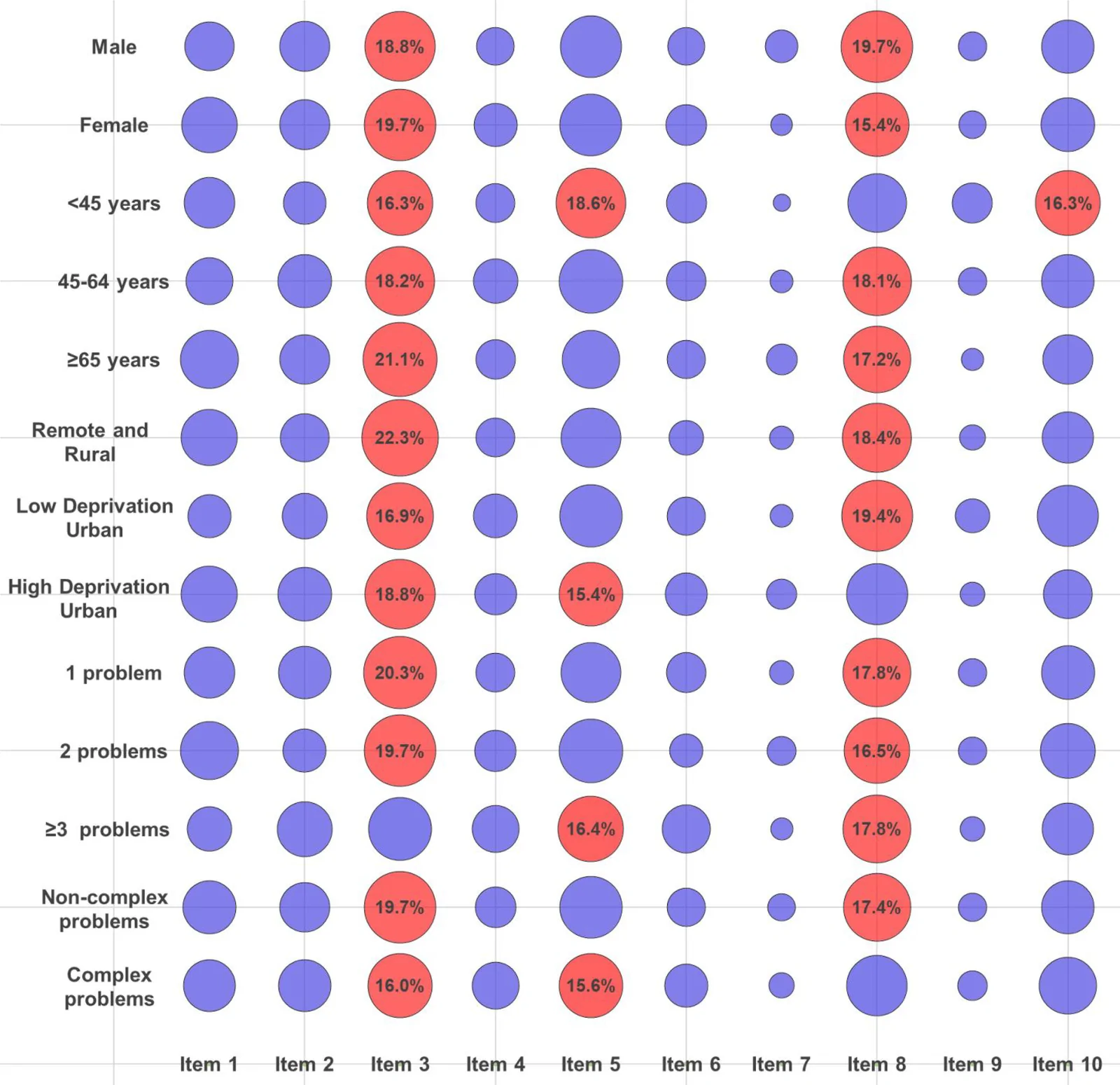
Bubble chart of the proportion of votes for each of the 10 CARE Measure items by patient and consultation characteristics. The size of each bubble corresponds to the percentage of patients who selected that item, with larger bubbles indicating higher selection rates. Red bubbles indicate the top two ranked items for each subgroup; blue bubbles indicate all other item selections*.*

Figure 3 shows the percentage of votes for each item across various sociodemographic subgroups, with red indicating the first and second ranked items. Across several subgroups, items 3 and 8 were the two most popular items. However, some subgroups differed, including patients aged <45 years (who prioritised item 5, followed by items 3, and 10 in equal second); those in HDU areas (items 3 and 5); those presenting with complex problems (items 3 and 5); and those presenting with ≥3 problems (items 8 and 5) (Figure 3).

### Two-item combinations (‘vote pairs’)

**Figure 4.**
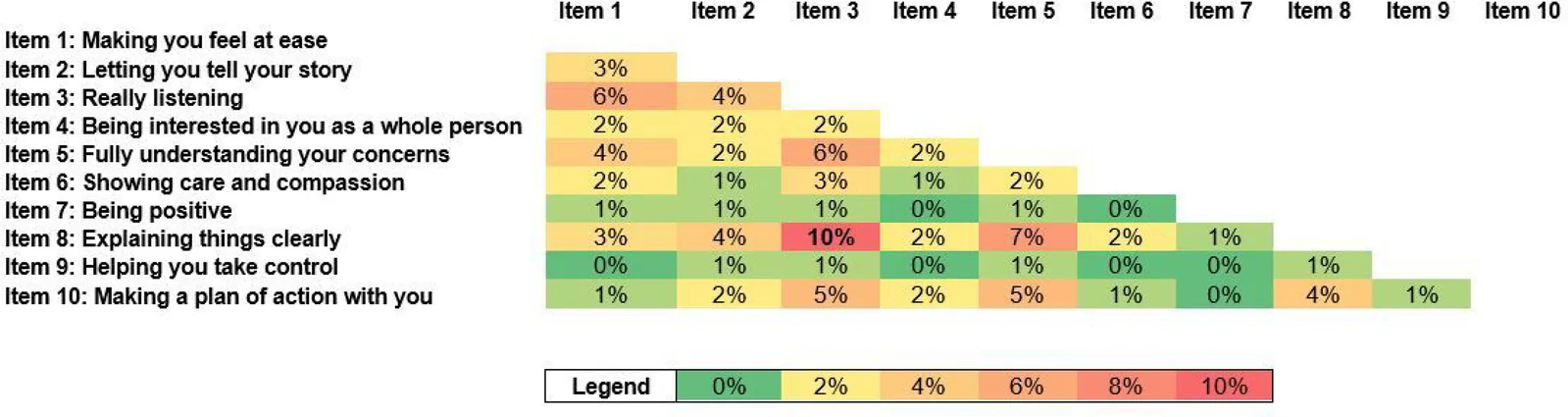
Heatmap of the proportion of patients selecting each two-item combination from the CARE Measure

Of the 1053 survey responses, 973 complete vote pairs were obtained (63 participants made no votes and 17 made only one vote). From the 45 possible two-item combinations, 43 were selected by at least one patient. The most popular two-item combination was items 3 and 8, selected by 10% of patients, with 90% selecting other pairs of items (Figure 4). In total, 52% of patients selected pairs that included either item 3 or item 8, while 38% selected pairs with neither of these items.

## Discussion

### Summary

Our study found that the optimal two-item version of the CARE Measure, according to psychometric evaluation, would consist of items 6 and 8 (‘Showing care and compassion’ and ‘Explaining things clearly’). This two-item combination had a correlation of 0.95 with the original 10-item version, compared with 0.81 for items 6 and 9, the combination selected by Takahashi *et al*.^7^ By contrast, patient preferences identified items 3 and 8 (‘Really listening’ and ‘Explaining things clearly’) as the two most important items, showing some divergence with the psychometric results. Furthermore, we found variations in item popularity and rank across individual and consultation characteristics (age, deprivation level, and consultation complexity) and low levels of agreement regarding the most popular two-item combination, with only 10% of patients selecting both items 3 and 8, and the vast majority (90%) choosing other combinations.

### Strengths and limitations

The strengths of this study were its relatively large sample size, the inclusion of patients from different regions of Scotland including urban deprived, urban affluent, and remote and rural areas. This study also replicated the same psychometric approach used to develop Japan’s two-item version of the CARE Measure,^7^ ensuring methodological consistency and comparability between studies. A limitation was its low patient response rate of 17% (12% HDU versus 27% LDU versus 20% RR). While it was initially planned to collect questionnaires within GP practices immediately after the consultation, where 70% response rates have been obtained,^14^ this was not possible owing to the COVID-19 pandemic. Nevertheless, the response rate is not dissimilar to the Scottish Government’s biennial national patient surveys.^17^

### Comparison with existing literature

This study is the first to explore patients' perspectives on the most valued aspects of empathy in GP consultations and to compare this with items selected on the basis of psychometric evaluation. These two approaches not only led to different conclusions from each other, but also from that of Takahashi *et al* ^7^ in the Japanese survey. The much higher pair-to-total correlation found for items 6 and 9 in Japan (0.98, ranked 1^st^) than in the UK (0.81, ranked 24^th^), emphasises the cross-cultural psychometric variability of survey items and necessity for caution.

While psychometric properties are important in survey development, they do not guarantee its validity across different contexts (for example, care settings, countries, or cultures) or population groups (for example, age or socioeconomic status).^18^ Cross-cultural differences in expressions of empathy within clinical settings have previously been demonstrated,^19–22^ and it would not be unexpected that the priorities differ between Japan and the UK. The cross-cultural translations of surveys can also affect how patients interpret, relate, and prioritise questions, as differences in language may alter meanings or omit concepts that do not directly translate.^23,24^

Moreover, existing literature has underscored the importance of incorporating patient views in the development of patient surveys to ensure measures are meaningful and relevant for those it intends to serve, thereby enhancing its quality and validity.^25,26^ The original 10-item CARE Measure was developed with patient feedback from affluent and deprived areas, confirming its face and content validity.^3^ Similarly, the paediatric Visual CARE Measure was adapted from the adult version with input from children and parents, resulting in three versions to ensure age-appropriate relevance and accessibility.^27^ These adaptations improved its usability, making the measure more meaningful and representative of the paediatric cohort, with its validity confirmed by feedback from children and their families.^28^ Previous studies have shown that patient surveys are more sensitive to patient experiences when developed with such input.^29–31^

### Implications for research and practice

These findings have broader implications for the development and abbreviation of patient-reported measures. While shortened measures can reduce response burden, there is a risk of compromising validity and measurement precision.^32^

To maintain validity, item-reduction should follow robust methodological guidance that integrates both psychometric performance and content relevance. Psychometric evidence generally supports retaining a minimum of three items per scale to ensure stable factor structures, although more may be needed to fully capture multidimensional constructs such as empathy.^33^ Importantly, this includes incorporating patient perspectives alongside statistical evaluation, in line with the COSMIN guidelines and ISOQOL standards.^10,11^ Our study reinforces the need for patient involvement in the adaptation of validated measures to ensure they remain accurate and meaningful. Moreover, the survey design provides limited insight into why patients prioritised certain items or how they interpret the relationships between them. Future qualitative research could explore these perspectives in greater depth, offering insights into the values and reasoning behind patient choices.

In conclusion, the optimal two-item version of the CARE Measure identified through psychometric evaluation in Scotland differs from both Scottish patients’ preferences and from the results of Takahashi *et al*’s^7^ equivalent psychometric evaluation in Japan. Moreover, there are variations in UK patient preferences and a low level of agreement regarding the most popular two-item combination. These findings suggest that reducing the 10-item CARE Measure to a two-item version may not be advisable.

## Data

The dataset relied on in this article is available from the corresponding author on reasonable request.
